# Feasibility of a real‐time dual energy markerless monitoring of lung tumors using a clinical room‐mounted stereoscopic and monoscopic x‐ray imaging system

**DOI:** 10.1002/mp.17966

**Published:** 2025-07-15

**Authors:** Zakary McLure, Chris Peacock, Mike Sattarivand

**Affiliations:** ^1^ Department of Physics & Atmospheric Sciences Dalhousie University Halifax Nova Scotia Canada; ^2^ Department of Medical Physics Nova Scotia Health Authority Halifax Nova Scotia Canada; ^3^ Department of Radiation Oncology Dalhousie University, Nova Scotia Cancer Centre Halifax Nova Scotia Canada

**Keywords:** markerless tracking, dual energy x‐ray imaging, stereoscopic/monoscopic IGRT

## Abstract

**Background:**

The motion of lung tumors during breathing poses challenges in stereotactic body radiotherapy (SBRT), warranting improved monitoring techniques. Breathing complicates SBRT by creating positional uncertainty in the lungs, traditionally managed with PTV margins, respiratory gating, or breath hold, each with significant drawbacks. While external and implanted markers for tracking have limitations, dual energy (DE) imaging offers a noninvasive, markerless solution that enhances soft tissue contrast and improves real‐time tumor localization accuracy and precision.

**Purpose:**

This study aims to develop a markerless real‐time DE tumor localization technique on a clinical room‐mounted x‐ray image guidance system to allow precise 3D stereoscopic and monoscopic lung tumor motion monitoring during radiotherapy.

**Methods:**

A motorized programmable breathing phantom combined with an anthropomorphic phantom was developed to simulate a lung tumor's respiratory motion, with various asymmetric 3D printed tumor models from lung patients. Tumor sizes ranged between 1.0 and 3.3 cm, with some having varying densities and imaged with varying doses. Real‐time images were acquired with a clinical ExacTrac stereoscopic imaging system at a rate of 1.67 Hz with high and low energies (140 and 60 kVp). Weighted logarithmic subtraction and an anti‐correlated noise reduction algorithm were used to generate DE images. Conventional single energy images (120 kVp) were acquired for comparison. Digital reconstructed radiographs from x‐ray imaging views were created to serve as templates for a template‐matching algorithm developed to localize tumor locations on x‐ray views. For the stereoscopic case where both imaging views were available, 3D triangulation was performed to localize the tumor. In the monoscopic case, when only one x‐ray view was available, the 3D tumor position was estimated using a single 2D localization, combined with a 3D probability density function (PDF) describing tumor motion.

**Results:**

Stereoscopic DE techniques demonstrated accurate localizations. The monoscopic view obstructed by the spine showed lower success rates than the view obstructed only by the rib bone. In stereoscopic cases, the localization success rates were similar (>96%) between single and DE techniques for large tumor sizes. As tumor sizes decreased, the localization success rates were higher for DE than the single energy technique showing an improvement of up to 25%. Monoscopic results demonstrated the same trend, with DE localization success rates improvement versus single energy by up to 53% for small tumors. DE showed more successful localization for less dense tumors by up to 60% compared to single energy. Tumors imaged with varying mAs values while remaining at optimal kVp settings showed similar localization success rates between single and DE techniques.

**Conclusion:**

A real‐time markerless tumor monitoring technique was developed utilizing a clinical room‐mounted stereoscopic/monoscopic image guidance system. DE increases the accuracy of successful tumor localization as compared to the conventional single energy approach, especially for smaller, less dense tumors. The use of PDFs may be a viable approach to monoscopic estimates when only one view is available.

## 1 INTRODUCTION

Lung tumors exhibit motions of up to 3 cm during a regular breathing cycle.^1^, ^2^ This is especially undesirable for cancer patients undergoing stereotactic body radiotherapy (SBRT), where large doses are delivered in a small number of fractions.^3^ Breathing motion creates positional uncertainty, which is traditionally managed by increasing planning target volume (PTV) margins, respiratory gating, or breath hold.^4^ The former increases unwanted dose delivery to nearby healthy organs at risk (OARs) as it requires large margins to define internal target volume (ITV).^5^ The latter two increase treatment time, require patient compliance, and can cause strain and discomfort to patients, especially those with respiratory conditions. Preferred approaches would involve direct real‐time monitoring of tumor motion.

Monitoring tumor motion can be performed through tracking body surface markers as a surrogate to internal target motion. However, the correlation between external markers and lung tumor motion has been shown to be unreliable due to intra‐fractional drift.^6^ A more direct technique uses implanted radio‐opaque markers on the target volume, but implanting these markers is invasive and can cause pneumothorax or hemorrhage.^7^ Additionally, studies have shown that the fiducial markers may drift more than 2.5 mm between fractions, affecting localization accuracy.^7^ Consequently, there is a growing demand for real‐time non‐invasive, markerless lung tumor monitoring.

Markerless tumor monitoring techniques have been explored,^8^, ^9^ notably those using template matching algorithms during treatment.^10^, ^11^ However, bony anatomy in the imaging field can hinder such localization tactics, which themselves rely on the high contrast between the tumor and surrounding tissue. The problem of tumor obstruction by bone can be addressed using dual energy (DE) imaging instead of conventional single energy (SE) techniques to suppress bone contrast and enhance soft‐tissue‐only images, thereby improving localization accuracy and precision.^11^, ^12^, ^13^, ^14^

Room‐mounted stereoscopic imaging systems such as ExacTrac (Brainlab AG, Germany) can be utilized to provide real‐time, sub‐millimeter accurate views of the treatment volume to monitor prostate motion using implanted fiducials,^12^ but they currently provide no reliable method for intra‐fraction lung tumor monitoring. Additionally, in a typical lung SBRT treatment, one of the two x‐ray imagers of the stereoscopic imaging system will be blocked by the rotating linac gantry during volumetric arc therapy (VMAT) treatment, hindering exact 3D location from a single 2D perspective. Developments in monoscopic (single view) localization of fiducial markers in prostate tumors for gantry mounted kV imaging systems offer a solution by correlating between motions in the anterior‐posterior and inferior‐superior directions to generate an informed estimate of the third dimension.^13^ This approach may also be extended to the context of lung tumors.

Montanaro et al. examined a variety of 2D‐3D inference methods in combination with on‐board SE 2D kV imaging systems with the goal of evaluating real‐time target tracking accuracies.^14^ A Gaussian PDF method, which models the probability density of the target,^15^ was investigated. Additionally, they examined arbitrary‐shape PDFs (A‐PDF), which represent the 3D target's probability density as a superposition of exponential functions based on previously observed positions.^16^ Furthermore, they explored interdimensional correlation (IDC), which leverages linear dependencies between left‐right lateral (LR), anterior‐posterior (AP), and superior‐inferior (SI) motions, exploiting the unambiguity of the SI component measured by the kV imager.^17^ Finally, they studied the Kalman filter, which combines measurements with a model based on prior knowledge to estimate system evolution over time.^18^, ^19^ Montanaro et al. found that for lung patients, Gaussian PDF proved to be the most accurate approach.

To our knowledge, there has not yet been a study that combines bone‐suppression from DE imaging with stereoscopic and monoscopic 3D localization for continuous lung tumor monitoring during treatment. The objective of this study is to develop a real‐time markerless tumor monitoring technique for stereoscopic imaging systems to monitor lung tumor motion during treatment, while also addressing the issue of intermittent monoscopic scenarios created by the rotating gantry.

## 2 MATERIALS AND METHODS

### 2.1 Data acquisition

#### 2.1.1 Phantom design and experiment setup

**FIGURE 1:**
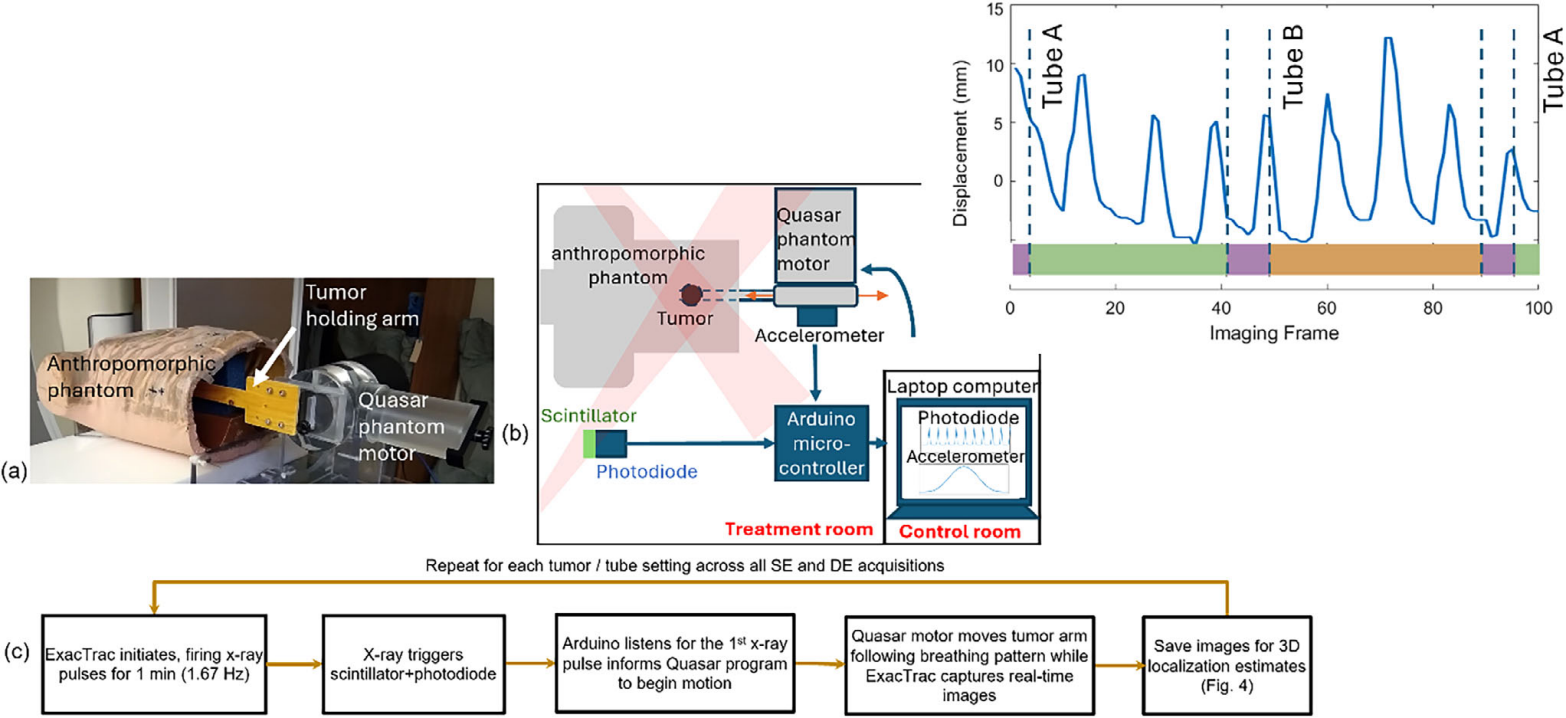
(a) The experimental phantom setup used for the real‐time motion experiment. (b) A schematic depicting the electronics used for the real‐time motion experiment. (c) Flowchart describing the imaging hardware interactions and sequences.

To emulate lung patient anatomy, an anthropomorphic breathing phantom (TBP, Mirion Technologies, New Jersey, USA) was used. Soft tissue thickness was increased by positioning a 5 cm thick plate of Lexan polycarbonate resin beneath the phantom. The built‐in expanding lung insert was removed, and an attached tumor model was inserted into the phantom's chest cavity (Figure 1a). Tumors were modeled using four 3D‐printed volumes from real lung patient data with maximum diameters of 1.0 , 2.0 , 2.6 , and 3.3 cm, and respective volumes of 0.4, 3.7, 4.3, and 17.6 cm^3^. Realistic lung tumor motion was achieved by repurposing a motor from a Quasar respiratory motion phantom (Modus Medical Devices, London, Ontario, Canada) and feeding to it a realistic typical lung motion (see Section 2.1.2). The Quasar phantom was mounted on a platform supporting the Lexan plate and breathing phantom at a fixed position on the treatment couch. The tumor holding arm was aligned to the SI direction, causing ∼2 cm motion in the SI direction from the applied breathing pattern (Figure 1b). The tumor arm was then inclined by 12° downward pitch to induce ∼5 mm tumor motion in the AP direction. Additionally, the couch was rotated by a 14° yaw angle to cause ∼5 mm tumor motion in the LR direction. Computed tomography (CT) images of each tumor (while at rest within the phantom) were acquired with a clinical CT simulator using the institution's lung SBRT imaging protocol (2.50 mm slice thickness). The CT scanner used was the GE Discovery CT590 RT with the specification and imaging parameters provided in Table 1.

**TABLE 1:** GE Discovery CT590 RT parameters.

Parameter	Value
Detector type	Solid‐state
No. of rows	16
Pixel spacing (mm)	1.27
Minimum slice thickness (mm)	0.625
Gantry aperture (cm)	80
Heat storage	8 MHU
mA range	10–800, 5 mA increments
Scan FOVs (cm)	50, 65 effective
kVp range	80, 100, 120, 135

#### 2.1.2 Real‐time imaging data acquisition

For each tumor size, the attached tumor model was aligned with the treatment room isocenter using the room lasers. Employing the Quasar motor motion program, the tumor followed a predefined trajectory based on input data containing typical lung breathing motion patterns. This data was sourced from the markerless lung target tracking AAPM Grand Challenge (MATCH)^20^ and used as a ground truth. Two sets of real‐time images were acquired for high and low energy sequences during two separate (but identical) breathing cycles and were subsequently used to generate DE images. To synchronize tumor motion with image acquisition, a photodiode (TCS34725, Adafruit Industries, New York, USA) coupled with 5 mm thick scintillating material (EJ‐208, Eljen Technology, Texas, USA) was positioned on the floor to detect x‐ray beam pulse (Figure 1b). A MATLAB script (MathWorks, Natick, MA) initiated tumor motion from a fixed start position upon detecting the first x‐ray pulse (Figure 1c). An accelerometer (SEN‐12786, SparkFun Electronics, Colorado, USA) attached to the tumor motion arm, outside of the field of view, measured the time delay between x‐ray pulse and tumor motion initiation. This time delay must be minimal to ensure synchronization of HE and LE images thus avoiding misregistration during DE image calculation. Both the accelerometer and photodiode communicated with an Arduino microcontroller (SparkFun, Colorado, USA) via I2C connector cables. The microcontroller combined the readings and transmitted them as a single byte of data via a USB cable to a laptop, where they were saved to a text file. The shaded pink regions in Figure 1b represent the stereoscopic x‐ray beams from Tube A and Tube B. Figure 1c demonstrates how these components interact as real‐time images are acquired. The detected motion delay, that is, the time delay between x‐ray detection and initiation of Quasar motion, was determined to be 110±7 ms (averaged over all energies), therefore the motor response time was considered negligible. To validate tumor motion against the MATCH breathing trajectory, a radio‐opaque ball bearing (BB) was attached to the tumor arm (Figure 1a). The BB trajectory on acquired images was calculated using 3D localization (Appendix I) and compared to the MATCH trajectory. The differences between the two trajectories averaged over 100 frames were 0.40±0.35 , 0.36±0.35 , and 0.35±0.34 mm for SE, HE, and LE acquisitions. Similarly, the differences between the BB on LE versus HE images were 0.20±0.24 mm. These values indicate synchronization between LE and HE to avoid misregistration as well as reproducibility of breathing cycles across various acquisitions.

For each of the four tumor sizes, real‐time stereoscopic DE and SE imaging series were acquired at an imaging frequency of 1.67 Hz using a clinical ExacTrac imaging system (Ver 6.2). The ExacTrac imager uses a pair of Varex RAD‐21 x‐ray tube and flat panel detectors. Details of the imaging system are provided in Table 2. Each series took 60 s to acquire and consisted of 100 imaging frames. The imaging parameters for the DE images were (140 kVp, 8 mAs) for high energy and (60 kVp, 25 mAs) for low energy acquisitions. These acquisition techniques were optimal DE parameters based on a previous study for lung patients to maximize tumor contrast in DE images, while ensuring that the total dose from DE does not exceed that of SE.^21^ The real‐time SE images were acquired using (120 kVp, 16 mAs) imaging parameters. This acquisition technique was chosen based on vendor recommendations for lung patient imaging.^22^

**TABLE 2:** ExacTrac imaging system parameters.

Parameter	Value
Resolution	512 × 512
Bit depth	16 bit
Pixel size	0.4 mm
FOV at isocenter	13.2 × 13.2 cm^2
Source to isocenter distance	343.5 cm
Isocenter to detector distance	218.5 cm
Detector area	(20.48 × 20.48) cm^2
Detector quantum efficiency	75% at 0 cycles/mm
Focal spot size	1.2 mm

In addition to varying tumor sizes, the effect of tumor density on localization was also investigated. The 2.6 cm tumor was 3D printed twice more with lower densities than the original by varying the printing infill parameter. In terms of HU (Hounsfield Units), within the tumor volume the densities that were 3D printed were 140 ±35 (the same density used in the size comparisons), ‐212 ±246, and ‐434 ±277 HU. Furthermore, the effect of varying mAs was investigated for the 2.6 cm tumor. In addition to the optimal DE acquisition parameters (HE: 140 kVp, 8 mAs and LE: 60 kVp, 25 mAs), the mAs values for both HE and LE were halved and doubled. Thus, the varying mAs that were used were 4 mAs and 16 mAs for HE, and 12.5 mAs and 50 mAs for LE image acquisitions. Similarly, the mAs values for the SE acquisitions were 8, 16, and 32.

### 2.2 Data analysis

#### 2.2.1 Dual energy image generation

For each frame in the real‐time image series, a soft‐tissue‐only DE image $I_{DE}^{ST}$ was calculated using a weighted log subtraction of the corresponding low energy image $I_{LE}$ from the high energy image $I_{HE}$ (Equation 1). Similarly, bone‐only DE images $I_{DE}^{B}$ were generated for use in an anti‐correlated noise reduction algorithm (ACNR)^23^ (Equation 2) which was applied to reduce noise in the soft‐tissue DE images.

(1) $$\begin{array}{c} \;\log\left(I_{DE}^{ST}\right)=\log\left(I_{HE}\right)\;-w_{s}\log\left(I_{LE}\right)\\ \;\log\left(I_{DE}^{B}\right)=-\log\left(I_{HE}\right)+w_{b}\log\left(I_{LE}\right) \end{array}$$

(2) $$\log\;\left(I_{DE,ACNR}\right)=\log\left(I_{DE}^{ST}\right)+w_{n}\log\left(I_{DE}^{B}\right)*h_{HPF}$$

Here, $h_{HPF}$ is a Gaussian high pass filter with a lower cutoff frequency of^24^ 0.2 pixel^‒1^. The noise suppression weighting factor $w_{n}$ was set to 0.9 for the calculation of all DE images.^24^ Similar to Hoggarth,^25^ the soft tissue DE weighting factor $w_{s}$ was chosen for each image by varying $w_{s}$ until bone suppression was maximized around the tumor. For Tube A images, this was accomplished by selecting regions of interest (ROIs) that encompassed bone (rib) and its surrounding soft tissue, then varying $w_{s}$ until the bone contrast‐noise ratio (CNR) was minimized. Similarly, the bone‐only DE weighting factor $w_{b}$ was chosen by varying $w_{b}$ to achieve the highest soft tissue suppression around the tumor while maximizing bone contrast. For the Tube B images, because of the spine obstructing the tumor, the previous approach was deemed challenging. Thus, a manual qualitative evaluation of the weighting factors was performed instead with the same approach of Tube A, that is, varying $w_{s}$ and $w_{b}$ until bone and soft tissue contrast around the tumor was minimized, respectively. The $w_{s}$ range was [1.16‐1.18] and [0.82‐1.35] for the two stereoscopic images from Tubes A and B respectively. A wider range of weighting factors were required for Tube B images compared to Tube A due to presence of thicker bone (spine) in view B compared to rib in view A. Similarly, the range for the bone‐only weighting factor $w_{b}$ was [2.10‐2.20] and [1.72‐2.00] for Tubes A and B, respectively.

#### 2.2.2 Template matching

**FIGURE 2:**
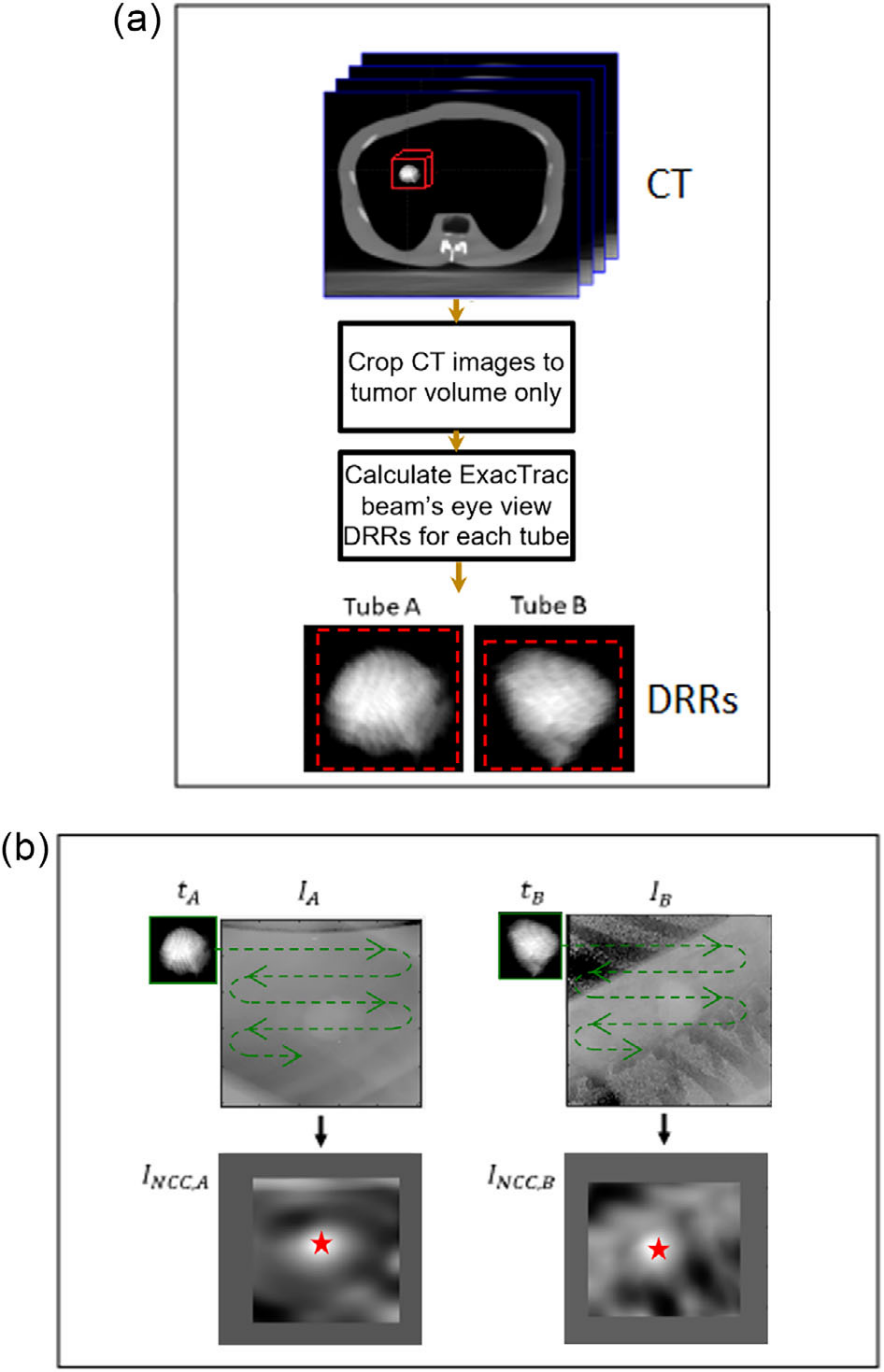
Description of the template matching procedure. (a) Template generation: Crop region around the tumor from CT and calculate DRR of the tumor for each stereoscopic imaging view. (b) Normalized cross‐correlation template matching using the DRR templates and the acquired SE or DE images. Identify and return the position of maximum correlation as the 2D location of the tumor for each view (red star).

Figure 2 describes the template matching procedure. The CT images of the tumors and phantom were exported to a commercial contouring and treatment planning system (Eclipse, Varian Medical Systems, Inc., Palo Alto, USA). Here, the gross tumor volume (GTV) was auto‐contoured using thresholding for each of the four tumor sizes from a CT with 2.5 mm slice thickness. Both the CT images and resulting contours were then exported in DICOM format from Eclipse to an in‐house MATLAB code, where tumor‐only CT images were generated by masking out the non‐tumor volume. Using a custom MATLAB algorithm which models the ExacTrac x‐ray source and detector geometry, tumor templates for each tumor size were created by generating digitally reconstructed radiographs (DRRs) using an algorithm from Siddon^26^ from each tumor‐only CT image, simulating the beam's eye views of the ExacTrac stereoscopic imaging system.^27^, ^28^ The tumor templates were then cropped from the DRRs with a two‐pixel margin (red dashed lines in Figure 2a) with the cropping region centered on the tumor's center of mass. These templates contain the same tumor shapes that are being tracked, as seen from the beam's eye view of the ExacTrac imaging system.

As per Mostafavi et al.,^10^ normalized cross correlation was used as a metric for template matching localization for each stereoscopic view. This algorithm was implemented using the MATLAB function *normxcorr2*, which calculates the normalized cross‐correlation between a template image t and a search image I using Equation 3:

(3) $$I_{NCC}\;\left(u,v\right)=\sum_{x,y}\frac{\left(I\left(x,y\right)-{\bar{I}}_{u,v}\right)\left(t\left(x,y\right)-\bar{t}\right)}{N\sigma_{I}\sigma_{t}}\;,$$

where $\bar{t}$ represents the average pixel value of the template, while ${\bar{I}}_{u,v}$ indicates the average pixel value within the template‐sized region of the image I, centered at pixel position (u,v). N denotes the total number of pixels in the template t. $\sigma_{t}$ and $\sigma_{I}$ represent the pixel standard deviation for the template and the sub‐image centered at position (x,y), respectively. To maintain the original image size within the cross‐correlation image $I_{NCC}$, I was padded with $N_{x}/2$ columns and $N_{y}/2$ rows of zeros. The global maximum position of $I_{NCC}$ for each stereoscopic projection image was selected to determine the tumor's location within each imaging view.

#### 2.2.3 Three‐dimensional tumor localization

**FIGURE 3:**
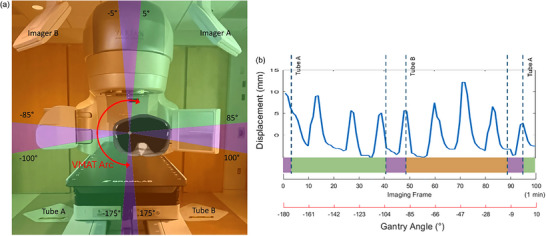
(a) Monoscopic (green/orange) and stereoscopic (purple) regions defined by the gantry angle during the treatment VMAT arc (red) of a lung SBRT patient. (b) Graph showing typical lung tumor motion during a 1‐min VMAT arc while real‐time imaging frames are being acquired during stereoscopic and monoscopic periods.

**FIGURE 4:**
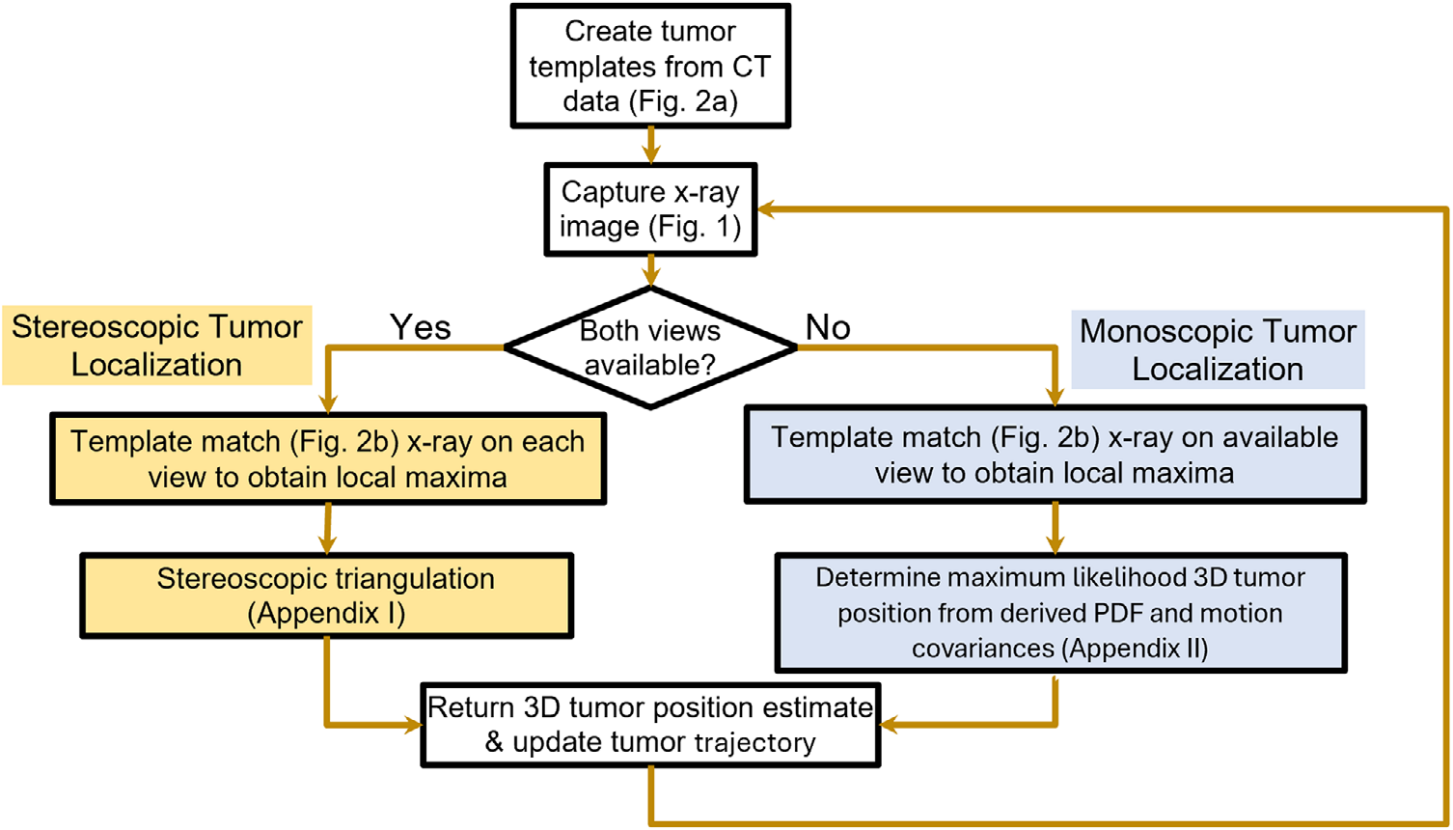
Flowchart describing 3D tumor localization. The stereoscopic and monoscopic approaches are described in Appendices I and II, respectively.

A typical lung SBRT treatment using volumetric arc therapy (VMAT) requires ipsilateral treatment arcs. This is illustrated in Figure 3 for a right lung case where gantry angle may range from ‐180° to 10°. The gantry motion during the treatment arc will cause real‐time ExacTrac imaging to go through periods of stereoscopic (where both views are available, i.e., the purple regions) or monoscopic (only one view is available, i.e., the orange or green regions) imaging. Figure 4 describes the workflow for image acquisition and 3D tumor localization depending on whether the gantry blocks one of the stereoscopic imaging views. In all scenarios, the template matching algorithm (described in Section 2.2.2) was used to locate the tumor in each 2D x‐ray image view. Stereoscopic triangulation, described in Appendix I, was used in cases where both imaging views were available. Monoscopic localization with a probability density function (PDF) approach, described in Appendix II, was used when only one imaging view was available.

Three‐dimensional localization positions were compared to the ground truth to calculate accuracy and precision. For each frame, a 3D localization was considered successful if the tumor was found within 3 mm of the ground truth. This choice was based on similar values in previous studies on lung SBRT tumor monitoring,^11^, ^20^, ^29^ given the inherent residual motion of lung tumors. The localization accuracy and precision along the anterior‐posterior, left‐right, and superior‐inferior directions were calculated using the mean and the standard deviation of the difference between the localized tumor position and the ground truth respectively.

## 3 RESULTS

**FIGURE 5:**
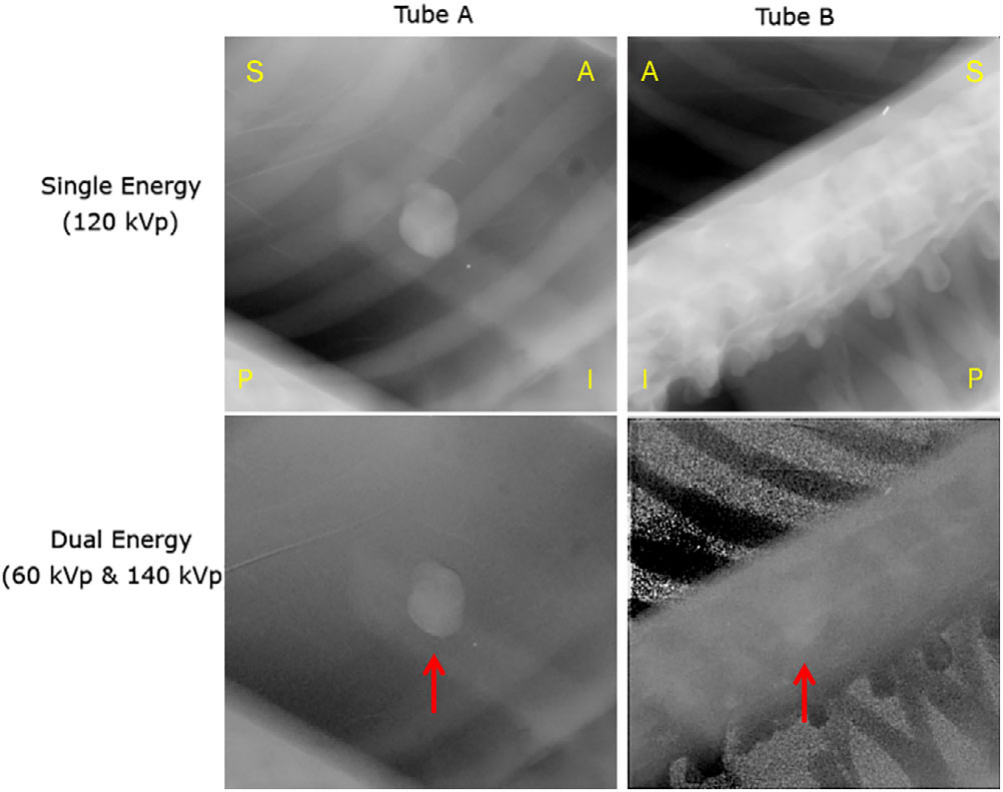
Dual energy and single energy images of a 2.6 cm tumor, as seen from both stereoscopic views. The left column is the monoscopic view from Tube A where the tumor is blocked by the rib. The right column is the monoscopic view from Tube B where the tumor is blocked by the spine. A/P and S/I represent the anterior‐posterior and superior‐inferior directions on the images.

Figure 5 shows examples of DE and conventional SE images for the 2.6 cm tumor from both stereoscopic views for one frame. From the perspective of Tube A, the moving tumor is often behind a rib. Likewise, for Tube B, the tumor is obscured for its entire trajectory by spinal vertebrae. The DE imaging, as shown in the bottom two images, allows for suppression of bone contrast in the region surrounding the tumor. As described in Appendix I, the stereoscopic imaging system results in images created by beams passing through the phantom at oblique incident angles (polar angle 48°, and azimuthal angles ± 45°).

**FIGURE 6:**
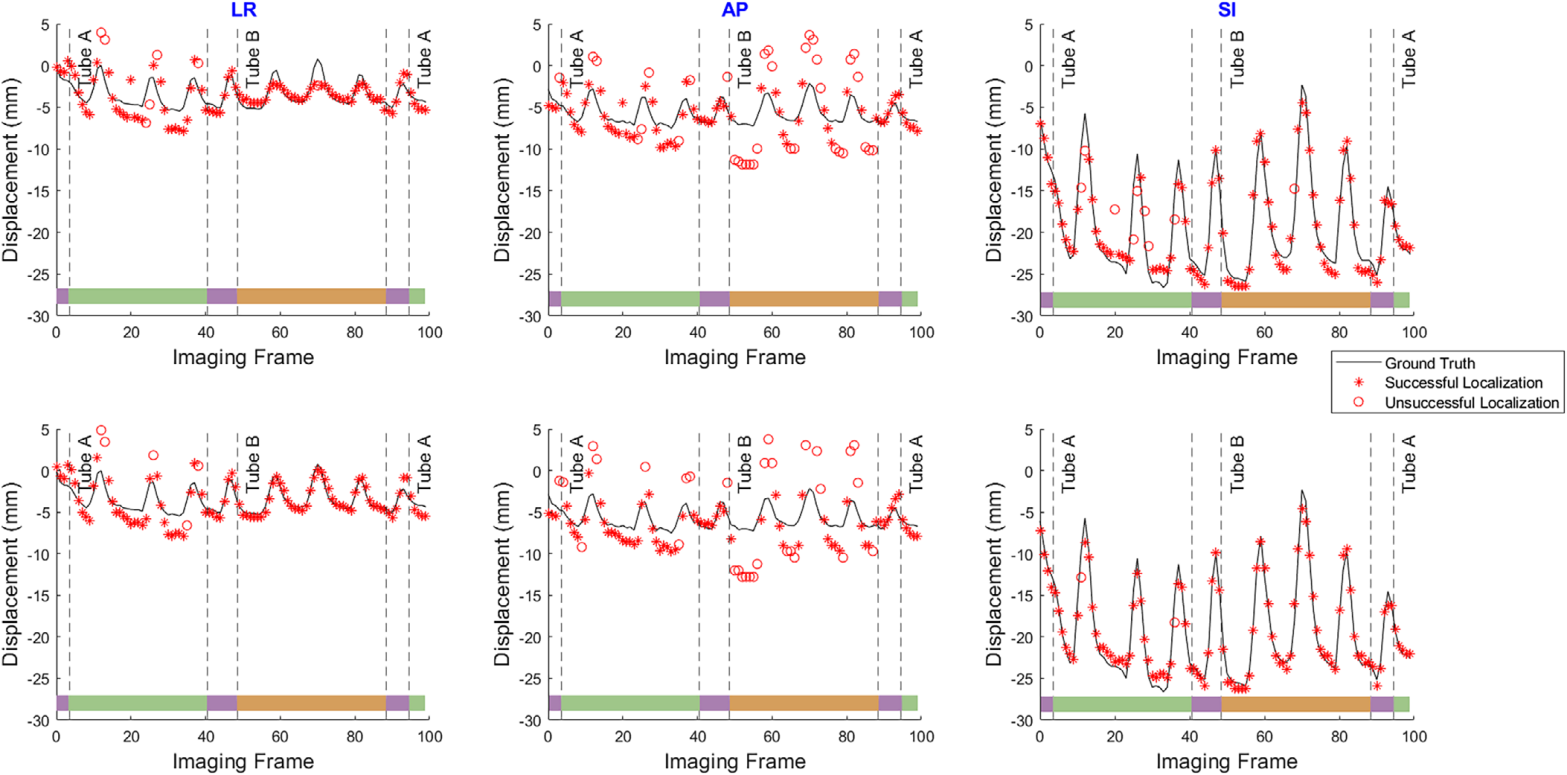
Estimated 3D tumor localizations using single and dual energy techniques for the 3.3 cm tumor. Asterisks indicate successful localizations (error < 3 mm), while empty circles indicate those that were unsuccessful. The color bars at the bottom of each graph label stereoscopic regions (purple), and monoscopic regions seen by Tubes A or B (green or orange respectively) as per Figure 3.

Figure 6 demonstrates results of the 3D tumor localization for both single and DE imaging techniques for the 3.3 cm tumor. Similar results were obtained for all other tumor sizes. The 3D localizations are plotted separately in left‐right lateral (LR), anterior‐posterior (AP), and superior‐inferior (SI) directions.

**FIGURE 7:**
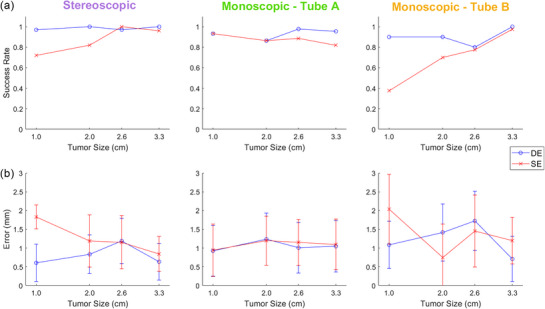
(a) Success rate and (b) error (accuracy ± precision) of stereoscopic localization, and monoscopic PDF estimates for each imaging view, with respect to tumor size, in the SI direction. Error bars are one standard deviation.

Figure 7a illustrates the localization success rates in the SI direction with respect to tumor size for both DE and SE techniques. The corresponding tumor localization accuracy and precision are demonstrated in Figure 7b.

For the two largest tumor sizes (3.3 cm, 2.6 cm), the localization success rate in the stereoscopic regions (when both imaging views are available) remained above 97% for DE, and above 96% for SE, indicating similar results between the two acquisition types. As tumor size further decreased, the comparison between DE and SE became distinctly noticeable, with the SE success rate decreasing to as low as 72% for the smallest tumor size (1.0 cm), while the DE success rate did not fall below 97% across all tumor sizes, indicating superior results than the SE technique.

In the monoscopic regions of Tube A, where the tumor was blocked by the ribs, the PDF estimate showed extremely similar results between SE and DE for the two smallest tumor sizes. However, it was different for the two largest tumor sizes, where DE once again exceeded SE's performance (98% vs. 82% success rates). In the monoscopic regions of Tube B, where the tumor was blocked by the spinal vertebrae, the DE technique showed a superior performance compared to the SE technique for the two smallest sizes of 2.0  and 1.0 cm. In terms of the accuracy of the localization (Figure 7b), the stereoscopic localization error generally remained around 1 mm, except for the smallest tumor size. Errors followed a similar trend for Tube A, while for Tube B, they were around 1.5 –2.0 mm. No statistical significance was observed in localizations errors between DE and SE techniques.

**FIGURE 8:**
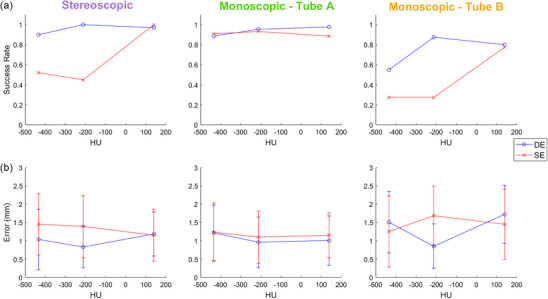
(a) Success rate and (b) error (accuracy ± precision) of stereoscopic localization, and monoscopic PDF estimates for each imaging view, with respect to tumor densities, in the SI direction. Error bars are one standard deviation.

Figure 8a shows the success rates of the tumors of varying densities. For the stereoscopic region, DE showed improved results over SE as density decreased, with a success rate difference of up to 55%. Tube A success rates were consistently above 88% for all densities for both DE and SE. Tube B success rates were considerably lower comparatively, yet DE still showed improvement of up to 60% compared to SE. Figure 8b illustrates the localization accuracies and precisions, where once again the errors ranged from 1  to 1.5 mm.

**FIGURE 9:**
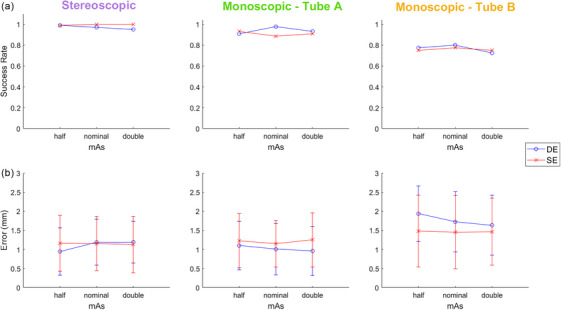
(a) Success rate and (b) error (accuracy ± precision) of stereoscopic localization, and monoscopic PDF estimates for each imaging view, with respect to varying tube mAs, in the SI direction. Nominal mAs represents vendor recommended settings, while mAs values corresponding to half and double those values are shown. Error bars are one standard deviation.

Figure 9a shows the success rates with respect to tube mAs, having nominal values representing mAs settings based on the literature and vendor recommendations,^22^ as well as half and double these nominal mAs values. For stereoscopic, and monoscopic Tube A and Tube B the results showed similar results for DE and SE across all mAs values. Likewise, the localization error values were all approximately between 1 and 2 mm.

## 4 DISCUSSION

Recent advancements in real‐time lung tumor tracking have included markerless imaging^30^ and data‐driven models^31^ to improve spatial and temporal accuracy during radiotherapy. Deep learning and AI‐driven prediction models have shown superior performance in tumor motion estimation compared to traditional methods.^31^ Markerless tracking using 2D kilovoltage imaging has shown potential as a non‐invasive approach for tumor localization.^30^ However, maintaining consistent and accurate tracking in clinical settings remains a challenge, requiring further ongoing development.

This study develops a real‐time markerless lung tumor monitoring technique with DE imaging capability using a clinical room‐mounted kV x‐ray imaging system. We addressed a significant issue associated with real‐time imaging during treatment using the imaging system, that is, the blocking of an x‐ray beam by the rotating linac gantry. This problem was addressed by applying a Gaussian PDF approach to calculate a 3D position estimate using only limited 2D information from single x‐ray view based on patient‐specific motion covariances of the lung tumor.

As per Figure 7, DE demonstrated similar success rates for the larger tumor sizes for both stereoscopic and monoscopic imaging techniques. SE success rates comparatively decreased toward the smaller tumor sizes of 2.0  and 1.0 cm. This illustrates that suppressing bone contrast in situations where lung tumors are obstructed by bone has a favorable effect on the tumor localization estimates for markerless tracking, especially when smaller tumor sizes are considered. This finding is consistent with previous DE studies using non‐stereoscopic systems.^25^, ^29^, ^32^, ^33^ The accuracy and precision of the stereoscopic localization reveals no noticeable trend for the larger tumor sizes, having errors on the order of ∼1 mm, while the largest uncertainties were observed for the 1.0 cm tumor. The significant overlap of the error bars, apart from stereoscopic readings for the 1.0 cm tumor, indicates there is little to no difference in localization errors between DE and SE approaches for stereoscopic imaging. This suggests that DE imaging reduces the number of image frames with large errors in tumor localization but has little effect in reducing the smaller localization errors brought on by imperfect bone tissue cancellation or errors in template matching. The accuracy and precision for both stereoscopic and monoscopic tumor localization were similar (1–2 mm) with no statistical significance between them. This is well within the PTV margin used in lung SBRT (typically 5 mm).

The localization of the smallest tumor (1.0 cm) was the least successful and generally with the highest errors. This is likely due to this tumor approaching a size comparable to neighboring (and overlapping) anatomical features such as the vertebrae along the phantom's spine. When the tumor overlaps with such features in Tube B, the signal from the cross‐correlation algorithm is not as strong, leading to a higher likelihood of incorrect localization. Considering that the GTV size in lung patients are typically larger than 1 cm due to accuracy limitation with small field dosimetry,^34^ the lack of detectability for the 1.0 cm tumor size may not in most cases pose a significant clinical problem. For a successful localization (success rate >80%) tumor sizes as low as 1.0 cm may be used with the DE technique, while the minimum tumor size should be >2.0 cm using the SE technique. When dealing with tumors nearing these thresholds or in case of significant bone obstruction, other considerations should be made such as use of DE or creating tumor template with thinner CT slices.

In general, DE outperforms SE for lower density tumors (Figure 8). Interestingly, in the stereoscopic and monoscopic Tube B cases, the success rates for the lowest density tumor did not further decrease from the middle density tumor (except for DE Tube B case). It is expected that tumor localization success rates monotonically reduce when tumor density is reduced. This unexpected result for the lowest density tumor (‐434 ± 277 HU) could be explained by the 3D printing process. The smallest density tumor contains prominent air cavities within the tumor structures, and thus the tumor edges are filled in to maintain structural integrity. The presence of air gaps in the structures is also reflected by the high HU standard deviation. These thicker and high‐density edges likely allow for the template matching algorithm to track the lowest density tumors and improve success rates. Future experiments will have to examine other methods of 3D printing that allow for the creation of more uniform lower density tumors.

The results of tumor localization in this study are comparable to previous reports. Patel et al.^29^ showed that with a 3 cm diameter tumor, DE fluoroscopy resulted in 99.1% of frames being tracked successfully, compared to 90.7% for SE. Unlike this study, their methodology used spherical tumors. Studies participated in the AAPM Grand Challenge (MATCH)^20^ reported success rates ranging from 38.9% up to 98%. This study differs from the linac‐mounted imaging approach by incorporating DE for bone suppression with stereoscopic localization, while calculating 3D tumor positions during monoscopic scenarios.

Due to the fixed geometry of the room‐mounted stereoscopic imaging system, tumor obstructions would be present throughout the entire treatment. This differs from tumor tracking procedures using on‐board imaging introduced in previous studies^32^, ^35^ where the imaging system revolves around the target volume and different bone obstructions occur at different parts of the patient's breathing pattern. Unlike the gantry‐mounted imaging system, the room‐mounted stereoscopic imaging system has the advantage of direct real‐time 3D localization of the tumor motion. Haytmyradov's study, which utilized a gantry‐mounted fast kV DE imaging technique, demonstrates that the poorest tracking accuracy occurs when the tumor is obstructed by the spine.^32^ In this study, 100% of the phases for the Tube B view dealt with spinal occlusion, while for Haytmyradov et al., it occurred one third of the time. Similar to this study, Haytmyradov et al. also demonstrated that the use of DE imaging improves the localization success rate when dealing with spinal overlap as compared to SE imaging. The effect of slice thickness on lung tumor localization using template matching was studied before. Haytmyradov et al. reported that for targets in excess of 10 mm, there is minimal difference among success rate, accuracy, and precision for different slice thicknesses ranging from 0.75  to 3.0 mm.^32^ It may be beneficial to use thinner slice thickness especially for SE imaging of smallest targets (5 mm) which are rare in lung SBRT. Thinner slice CT imaging in lung treatment is usually not used in clinics to minimize lung tumor contouring during planning process. In this study, slice thickness of 2.5 mm was utilized based on our clinical CT scanning protocol for lung patients. Thinner slice thickness for tumor template creation is likely to improve localization for smaller tumors in challenging cases such monoscopic Tube B.

Figure 9 indicates similar results of tumor localization success rate for the three mAs settings. Theoretically, mAs has no effect on tumor contrast but affects quantum noise for SE technique. Thus, for a quantum noise limited system, the tumor CNR theoretically increases or decreases by square 2 factor when mAs doubles or halves, respectively. While it is not expected these factors linearly translate to tumor localization success rates, a wider range change in mAs (than a factor of two) could likely affect the results. Larger mAs values (at the cost of more dose) will likely improve localization accuracy for smaller tumors. For SE acquisition, the nominal mAs values were vendor recommended imaging techniques based on treatment site (lung) and patient size.^22^ For the DE acquisition, nominal mAs values were optimized values for the ExacTrac imaging from a previous study^21^ to ensure maximum lung tumor contrast is achieved while imaging dose does not exceed that of SE acquisition.

Real‐time imaging adds dose to the PTV and surrounding OARs which cannot be ignored. Long‐term effects of imaging dose raise concern especially for pediatric patients. Imaging dose strongly depends on imaging device and previous studies quantified it for specific radiotherapy image guidance systems and for various treatment sites. Bowman^21^ reported that with the optimized DE acquisition parameters (which we used here) the surface dose per frame from the ExacTrac system for lung imaging technique was 0.52 mGy using both single and optimized DE techniques. Abeywardhana et al. quantified the patient‐specific imaging dose for ExacTrac monoscopic/stereoscopic real‐time kV image guidance received by lung and prostate patients.^36^ With a validated Monte Carlo model using DOSEXYZnrc,^37^ they found that bone and skin received the highest imaging dose, with larger patients having received more dose in these regions than smaller ones. For 30 lung patients, the dose delivered to 2% of organ volume (D2) of the bone and skin maximized at 4.30% (2062 mGy) and 1.98% (951 mGy) of the prescription dose respectively. Additional imaging dose to the PTV itself was reported to be 2.42% of the prescription. These values are below the AAPM‐TG‐180 recommended imaging dose threshold of 5%, beyond which imaging dose must be accounted for during the treatment planning process.^38^ In a follow‐up study quantifying how imaging doses could affect OAR constraints in treatment planning, Abeywardhana investigated the effect of additional dose from monoscopic/stereoscopic real‐time tumor monitoring in 30 lung SBRT patients with 535 OAR constraints.^39^ However, there was only one instance out of those 535 constraints (0.2% of cases) that led to an OAR dose constraint failure when adding imaging dose to treatment dose, in which the imaging dose was 1.9% of the prescription. In all cases, the real‐time imaging dose did not exceed the recommended TG‐180 threshold, suggesting that additional real‐time imaging dose may not be clinically significant. Moreover, a reduction of PTV margins via real‐time imaging (e.g., by minimizing ITV margins to only residual motion) could significantly reduce nearby OAR treatment doses, a fact which was not accounted for in the above imaging dose studies.

The methodology and results of DE imaging in this study may also be applied to other stereoscopic image guidance systems such as CyberKnife,^40^, ^41^ Vero system,^42^ and SyncTracX,^43^ where frequent blocking of one view is less of a concern. Combining the stereoscopic and monoscopic techniques described throughout this paper, the clinical room‐mounted image guided system could be capable of real‐time lung tumor monitoring with the improved success rate brought on by DE imaging techniques. Specific hardware and software adaptation may be required for each system to implement real‐time DE acquisition and analysis, for example, fast kV switching, multi‐layer detectors, increase heat load of x‐ray tube for real‐time acquisition, implementation of 3D tumor localization algorithm, etc. Clinical workflow may also be affected, for example, identifying lung patients with least tumor bone obstruction. This capability needs to be evaluated on lung cancer patients in clinical settings to facilitate clinical adaptation.

This work is a proof‐of‐principle study for DE real‐time lung tumor tracking image guidance, since the current version of ExacTrac lacks fast kV switching for DE image acquisition, while the real‐time SE acquisition does not have this hardware limitation. Fast kV switching is a well‐developed technology which has been used in previous studies to track lung tumor in real‐time for image guidance applications and spectral imaging.^32^, ^44^ An alternative approach for dual‐energy image acquisition involves the use of multi‐layer detectors, which enables simultaneous capture of high‐ and low‐energy projection data within a single exposure.^45^ This method can mitigate the temporal mismatches associated with sequential acquisition and has demonstrated promise in cone‐beam computed tomography (CBCT), for quantitative dual‐energy imaging, beam hardening reduction,^44^ and improving CT number uniformity.^46^ The current ExacTrac system allows real‐time imaging with a frequency of 1.67 Hz (i.e., 100 images per minute). While 1.67 Hz was used in this study and previous prostate real‐time image guidance studies,^12^, ^13^ this could be a limitation as most previous studies in real‐time lung imaging used a higher frequencies in the range of 5–10 Hz.^1^, ^20^ Nevertheless, the preliminary results indicate that the approach used in this study show promise and could sufficiently improve given higher imaging frequencies. As per previous studies,^11^, ^47^ we assumed rigid tumor motion during breathing cycle which could also be a limitation of the study. However, the dynamic nature of tumors during the breathing cycle may lead to changes in tumor shape and size during the breathing cycle for certain patients. Fortunately, this information is available before patient's treatment from planning 4DCT thus it can be incorporated into template matching tumor localization. For instance, separate templates can be calculated from specific points of breathing cycle (e.g., 0%, 50%, etc.) and used during real‐time tumor monitoring. The computation requirement for tumor localization was 70 s (0.7 s per frame) for 1 min real‐time acquisition using Intel Core i7 @ 2.2 GHz CPU. Feasibility of implementation for real‐time localization can be achieved using GPUs or faster computer languages (such as C instead of MATLAB).

Future clinical implementation of dual‐energy imaging for lung tumor tracking will require several advancements beyond the scope of this study. The use of fast kVp switching or multi‐layer detector as described previously offers a hardware solution for DE image acquisition. Adaptive and automated weighting factor updating which has been reported previously^48^, ^49^ is also essential for real‐time DE imaging. While some aspects of this work relied on manual adjustments, automation will be critical for streamlining clinical workflows and removing operator dependency. MLC tracking, as outlined in TG‐264,^50^ allows for the integration of real‐time target monitoring with treatment delivery systems. These developments, alongside robust clinical trials, will be vital to transition markerless DE imaging systems from experimental setups to routine clinical practice.

## 5 CONCLUSION

A real‐time markerless imaging system was developed, combining stereoscopic and monoscopic tumor tracking techniques for lung tumor patients. Integration of DE significantly improves tumor localization success rates compared to the conventional SE approach, particularly for smaller tumors. PDFs provide a promising approach to monoscopic estimates when the room‐mounted stereoscopic imaging system is partially blocked by the rotating gantry. This study indicates the potential for accurate real‐time markerless tumor tracking, which could improve precision radiotherapy by removing the need for a large ITV margin and thus minimizing treatment of healthy tissues.

## CONFLICT OF INTEREST STATEMENT

The authors have no relevant conflicts of interest to disclose.

## Figures and Tables

**FIGURE A1:**
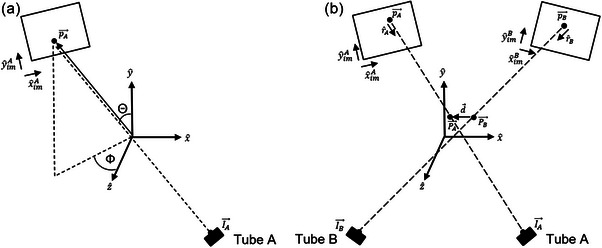
a) ExacTrac detector geometry polar angle *θ* and inclination Φ, with respect to treatment room coordinates, using the ray‐traced line between view A and Tube A. b) Illustration of how triangulation is performed by determining the shortest crossing line between a pair of vectors traced back from image coordinates to x‐ray tubes.

**FIGURE A2:**
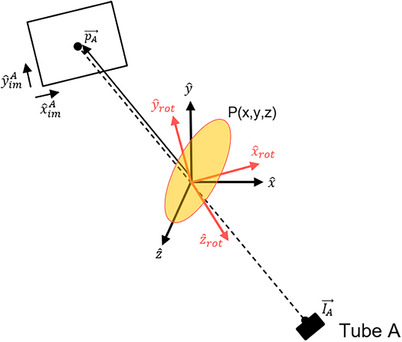
Monoscopic localization uses a Gaussian PDF derived from motion covariances to determine the maximum likelihood position (orange ellipsoid) along the ray‐traced line between the x‐ray source $\vec{I_{A}}$ and the 2D imager point $\vec{p_{A}}$ obtained from template matching. Lung motion is most prevalent in the SI (z) direction.

## APPENDIX I

### Stereoscopic localization using 3D triangulation

Adopting a stereoscopic triangulation methodology akin to Wei et al.^51^ and Brost et al.^52^ the 3D localization of the tumor was determined by identifying the shortest line segment connecting two back‐traced rays, which originate from a pair of 2D tumor localizations (determined using template matching as per Section 2.2.2). Referring to Figure A1a with known the orientation (polar angle *θ* and azimuthal angle Φ) and inter‐pixel spacing for each imaging panel i, the relationship between the 2D position of a pixel on the panel $\left(x_{im},y_{im}\right)$ and the corresponding point on room 3D coordinates ${\vec{p}}_{i}$ is expressed by the following equation:

(A1)
$${\vec{p}}_{i}=R\cdot \left[\begin{array}{c} x_{im}^{i}\\ y_{im}^{i}\\ 0 \end{array}\right]\cdot \frac{SDD}{SAD}$$

where SDD (source to detector distance) and SAD (source to axis/isocenter distance) are used as a scaling factor, and $R=R_{x}\;\cdot R_{y}$ is a matrix used to rotate the detector coordinate system to the room coordinate system. The rotation matrices $R_{x}$ and $R_{y}$ are defined in Equation A2.

(A2)
$$\begin{array}{cc} R_{x} & =\left[\begin{array}{ccc} 1 & 0 & 0\\ 0 & \cos\left(90^{\circ }-\theta \right) & -\sin\left(90^{\circ }-\theta \right)\\ 0 & \sin\left(90^{\circ }-\theta \right) & \cos\left(90^{\circ }-\theta \right) \end{array}\right]\;,\\ & \;\;R_{y}=\left[\begin{array}{ccc} \cos\left(\Phi \right) & 0 & \sin\left(\Phi \right)\\ 0 & 1 & 0\\ -\sin\left(\Phi \right) & 0 & \cos\left(\Phi \right) \end{array}\right]\; \end{array}$$

For ExacTrac imaging panels A and B, $\theta_{A}=\;\theta_{B}=48^{\circ }$, $\Phi_{A}=-45^{\circ }$, and $\Phi_{B}=45^{\circ }$. Likewise, the SDD and SAD are 343.5  and 218.5 cm, respectively.^28^ Given a pair of 2D image coordinates $\left[x_{im}^{A},y_{im}^{A}\right]$ and $\left[x_{im}^{B},y_{im}^{B}\right]$ obtained from template matching applied to each of the x‐ray images, points ${\vec{p}}_{A}$ and ${\vec{p}}_{B}$ can be derived. The triangulation process proceeds by using the pair of vectors between the x‐ray sources and their respective panel detectors to determine the most probable 3D position where the pair of back‐traced rays would intersect (Figure A1b). ${\vec{P}}_{A}$ and ${\vec{P}}_{B}$ represent the points on the source‐detector ray, defining the shortest cross‐line distance between the two rays. These points are determined by scaling the unit vector for each source‐detector pair ${\hat{r}}_{A}$ and ${\hat{r}}_{B}$ by scalars $L_{A}$ and $L_{B}$, originating from the pixel positions ${\vec{p}}_{A}$ and ${\vec{p}}_{B},$ respectively:

(A3)
$$\begin{array}{c} \;{\vec{P}}_{A}=L_{A}\;{\hat{r}}_{A}+{\vec{p}}_{A}\\ \;{\vec{P}}_{B}=L_{B}\;{\hat{r}}_{B}+{\vec{p}}_{B} \end{array}$$

where ${\hat{r}}_{A}=\frac{{\vec{I}}_{A}-{\vec{p}}_{A}}{|{\vec{I}}_{A}-{\vec{p}}_{A}|}$, ${\hat{r}}_{B}=\frac{{\vec{I}}_{B}-{\vec{p}}_{B}}{|{\vec{I}}_{B}-{\vec{p}}_{B}|}$, and ${\vec{I}}_{A}$ and ${\vec{I}}_{B}$ are source points on Tube A and B, respectively. The proposed method suggests the most probable 3D ray coincidence point to be the midpoint along the shortest line segment between the two rays. This midpoint is determined by locating the halfway point along a line connecting the two rays and perpendicular to both. The shortest line segment $\vec{d}$, connecting points ${\vec{P}}_{A}$ and ${\vec{P}}_{B}$, is computed by projecting the vector connecting points ${\vec{p}}_{A}$ and ${\vec{p}}_{B}$ onto a unit vector that is perpendicular to both ${\hat{r}}_{A}$ and ${\hat{r}}_{B}$:

(A4)
$$\vec{d}=\left(\left({\vec{p}}_{A}-{\vec{p}}_{B}\right)\cdot \frac{{\hat{r}}_{A}\times {\hat{r}}_{B}}{\left|{\hat{r}}_{A}\times {\hat{r}}_{B}\right|}\right)\;\frac{{\hat{r}}_{A}\times {\hat{r}}_{B}}{\left|{\hat{r}}_{A}\times {\hat{r}}_{B}\right|}$$

The scaling coefficients $L_{A}$ and $L_{B}$ for each of the back‐trace unit vectors are derived using Equations A3 and A4 as follows, with the knowledge that ${\vec{P}}_{A}={\vec{P}}_{B}+\vec{d}$:

$$L_{A}{\hat{r}}_{A}+{\vec{p}}_{A}=L_{B}\;{\hat{r}}_{B}+{\vec{p}}_{B}+\vec{d}$$

$$L_{A}{\hat{r}}_{A}-L_{B}\;{\hat{r}}_{B}={\vec{p}}_{B}\;-{\vec{p}}_{A}+\vec{d}$$

$$L_{A}\left({\hat{r}}_{A}\times {\hat{r}}_{B}\right)-L_{B}\;\left({\hat{r}}_{B}\times {\hat{r}}_{B}\right)=\left({\vec{p}}_{B}-{\vec{p}}_{A}+\vec{d}\right)\;\times {\hat{r}}_{B}$$

$$\;L_{A}=\frac{\left|\left({\vec{p}}_{B}-{\vec{p}}_{A}+\vec{d}\right)\times {\hat{r}}_{B}\right|}{\left|{\hat{r}}_{A}\times {\hat{r}}_{B}\right|}$$

A similar approach is used to isolate $L_{B}$. The scaling coefficients are therefore:

(A5)
$$\begin{array}{c} \;L_{A}=\frac{\left|\vec{D}\;\times \;{\hat{r}}_{B}\right|}{\left|{\hat{r}}_{A}\;\times \;{\hat{r}}_{B}\right|}\;\\ \;L_{B}=\frac{\left|\vec{D}\;\times \;{\hat{r}}_{A}\right|}{\left|{\hat{r}}_{B}\;\times \;{\hat{r}}_{A}\right|}\; \end{array}$$

where $\vec{D}={\vec{p}}_{B}\;-{\vec{p}}_{A}+\vec{d}$. The center point between A and B is calculated as the average of the two points $\vec{v}=\frac{\;{\vec{P}}_{A}+{\vec{P}}_{B}}{2}$.

## APPENDIX II

### Monoscopic Localization

The use of a Gaussian probability density function (PDF) to estimate a 3D position in monoscopic imaging using on‐board imagers is described by Poulsen et al.^53^ and was replicated by Stevens et al. for the ExacTrac system in the context of prostate tumors.^12^ The method depicted in Figure A2 requires a priori information in the form of motion covariances Q. These covariances can either be derived from the patient‐specific real‐time tumor localization before treatment, or from established patient population averages. The latter is expected to be less accurate, as it represents the population average. Therefore, in this study, patient‐specific covariances were calculated using the tumor motion estimated by stereoscopic localization from all 100 frames as per Appendix I.

(A6)
$$Q=\left[\begin{array}{ccc} var_{x} & cov_{xy} & cov_{xz}\\ cov_{xy} & var_{y} & cov_{yz}\\ cov_{xz} & cov_{yz} & var_{z} \end{array}\right]=\left[\begin{array}{ccc} 0.0695 & 0.0281 & 0.1929\\ 0.0281 & 0.0273 & 0.1734\\ 0.1929 & 0.1734 & 19.3994 \end{array}\right]\;mm^{2}$$

By using this covariance matrix, the PDF for the tumor positions P(x,y,z) can be determined in a coordinate system rotated to align with the imaging plane, but with the same origin as the patient coordinate.^54^ The inverse of the rotation matrix from Appendix I, $R=R_{x}\;\cdot R_{y}$, is applied to the rotated 3D position vector  $\;{\vec{r}}_{rot}=\left[x_{rot},y_{rot},z_{rot}\right]$ to determine the 3D room position ${\vec{p}}_{i}$,

(A7)
$${\vec{p}}_{i}=R^{-1}\;\cdot {\vec{r}}_{rot}$$

where i = A for Tube A and i = B for Tube B. The Gaussian PDF in the rotated coordinate system aligned with the imaging plane is described by

(A8)

Representing the elements of the rotated covariance matrix as

(A9)
$$R^{-1}Q^{-1}R=\left[\begin{array}{ccc} A_{rot} & D_{rot}/2 & E_{rot}/2\\ D_{rot}/2 & B_{rot} & F_{rot}/2\\ E_{rot}/2 & F_{rot}/2 & C_{rot} \end{array}\right]\;,$$

the expected value of the position along the axis normal to the imaging plane $\left\langle z_{rot}\right\rangle$ is^27^

(A10)
$$\begin{array}{cc} \langle z_{rot}\rangle & =SAD\left[A_{rot}{\left(\frac{x_{im}}{SDD}\right)}^{2}+B_{rot}{\left(\frac{y_{im}}{SDD}\right)}^{2}\right.\\ & \;\left.+D_{rot}\frac{x_{im}y_{im}}{SDD^{2}}-E_{rot}\frac{x_{im}}{2\cdot SDD}-F_{rot}\frac{y_{im}}{2\cdot SDD}\right]\sigma^{2}, \end{array}$$

where SDD represents the source‐to‐detector distance and SAD is the source‐to‐isocenter distance, both of which are described in Appendix I. Sigma represents the standard deviation, found using

(A11)
$$\begin{array}{cc} \sigma & =\left[A_{rot}{\left(\frac{x_{im}}{SDD}\right)}^{2}+B_{rot}{\left(\frac{y_{im}}{SDD}\right)}^{2}+C_{rot}\right.\\ & \;{\left.+D_{rot}\frac{x_{im}y_{im}}{SDD^{2}}-E_{rot}\frac{x_{im}}{SDD}-F_{rot}\frac{y_{im}}{SDD}\right]}^{-\frac{1}{2}}\;. \end{array}$$

Adjusting the image coordinates $\left[x_{im},y_{im}\right]$ based on this expectation value allows for the determination of the (rotated) 3D position of the lung tumor ${\vec{r}}_{rot}=\left[x_{rot},y_{rot},z_{rot}\right]$

(A12)
$$x_{rot}=x_{im}\;\frac{SAD-z_{rot}}{SDD}$$

(A13)
$$y_{rot}=y_{im}\;\frac{SAD-z_{rot}}{SDD}$$

Finally, we use the inverse rotation matrix, shown with Equation A7, to calculate the 3D monoscopic coordinates.
